# Synthesis, Characterization, and Photocatalytic Properties of Bamboo Charcoal/TiO_2_ Composites Using Four Sizes Powder

**DOI:** 10.3390/ma11050670

**Published:** 2018-04-25

**Authors:** Bin Wang, Bo Liu, Xing-Xiang Ji, Ming-Guo Ma

**Affiliations:** 1 Engineering Research Center of Forestry Biomass Materials and Bioenergy, Beijing Key Laboratory of Lignocellulosic Chemistry, College of Materials Science and Technology, Beijing Forestry University, Beijing 100083, China; wangbin7902@163.com; 2 State Key Laboratory of Biobased Material and Green papermaking, Qilu University of Technology (Shandong Academy of Sciences), Jinan 250353, China; xxjt78@163.com

**Keywords:** composites, bamboo charcoal, TiO_2_, photocatalytic activity

## Abstract

Visible-light-active bamboo biochar/TiO_2_ composites were fabricated by the calcination method using C_16_H_36_O_4_Ti as the titanium source and bamboo powder with different sizes as the carbon source. The TiO_2_ nanoparticles were observed to disperse onto the surface of bamboo biochar fiber. The sizes of the bamboo powder played an important role in the microstructures and the properties of bamboo biochar/TiO_2_ composites. The bamboo biochar/TiO_2_ composites displayed the photocatalytic activities both under visible light irradiation and UV irradiation. The adsorption isotherms better fitted Freundlich isotherm models and the photodegradation reactions followed pseudo-first-order kinetics. Bamboo charcoal/TiO_2_ composites exhibited high stability after up to four cycles. This research could pave the way for high-value applications of biomass in the environmental field.

## 1. Introduction

Recently, photocatalytic composites have attracted much attention due to their applications potential in the environmental fields. Among of various composites, the carbon-based composites displayed the advantages of stable, highly electroconductive, and easy to be modified [1]. Until now, there are a few reports on the preparation of the carbon-based composites, including carbon/tungsten acid bismuth [2], carbon/iron and manganese oxides [3], carbon/zinc oxide [4], carbon/graphitic carbon nitride [5], carbon-based quantum dots [6], etc.

It is well known that TiO_2_ is one of semiconductor photocatalysts with suitable band gap, high biological stability, and excellent photocatalytic properties [7,8]. It is well known that TiO_2_ has a wide band-gap of 3.2 eV in the ultraviolet light zone, limiting its applications in the visible light band. Many strategies are applied to change the light absorption ability of TiO_2_, such as dye sensitizing [9], ion doping [10], heterojunction [11], etc. It is reported that the C-doped TiO_2_ displayed the increase photocatalytic activity in the visible light region [12]. Mohamed et al. synthesized the C-TiO_2_ thin films via the sol-gel method using the Tween 80 both as the directing agent and the carbon source [12]. Wu et al. prepared the C-TiO_2_ composites with the narrow band gap and the optical property by the solvothermal method combining the calcination [7]. Yao et al. used different calcination temperatures to prepare the C-doped TiO_2_ nanoparticles with the high photocatalytic performance at 400 °C [13]. Zhang et al. explored in situ create the C-doped hollow TiO_2_ spheres with a superior photocatalytic activity [14].

Bamboo is one of typical biomass, which can be used as a carbon source. Bamboo-based carbon materials have wide applications due to their strong adsorption ability for organic pollutants and metal ions [15]. Bamboo charcoal is an economical carbon during the preparation procedure, when compared with the other carbon precursors (graphene, carbon nanotubes, and fullerene) [16]. Chou et al. prepare the bamboo charcoal/TiO_2_ composites in a high-efficiency dye sensitized solar cell [17,18]. Vignesh et al. synthesized the bamboo charcoal/TiO_2_ composite with plasma surface treatment by the sol-gel method [19]. Recently, Qian et al. produced the biomorphic charcoal/TiO_2_ composites using the moso bamboo as templates [20]. However, as far as we know, there has been little report on the synthesis of the bamboo charcoal/TiO_2_ composites in the environmental field.

In this article, the bamboo charcoal/TiO_2_ composites were synthesized by the calcination route using the C_16_H_36_O_4_Ti as the titanium source and the bamboo powder as the carbon source. The synergistic effects of carbon doping with TiO_2_ were explored in detail. The photocatalytic activities of the bamboo charcoal/TiO_2_ composites were investigated under the visible light irradiation and UV irradiation.

## 2. Experimental Section

### 2.1. Materials

All of the as-received chemicals were of analytical grade, which were used without further purification. All of the experiments were done under the air atmosphere. The dragon bamboo was selected from Yunnan region, China, and was collected as raw material for the synthesis of the samples. Both the tetrabutyl titanate (C_16_H_36_O_4_Ti, Xingjin, 98.5%, Beijing, China) and potassium hydroxide (KOH, Beijing Chemical Works, Beijing, China) were obtained in China.

### 2.2. Preparation Process of Bamboo Charcoal/TiO_2_ Composites

**Figure 1:**
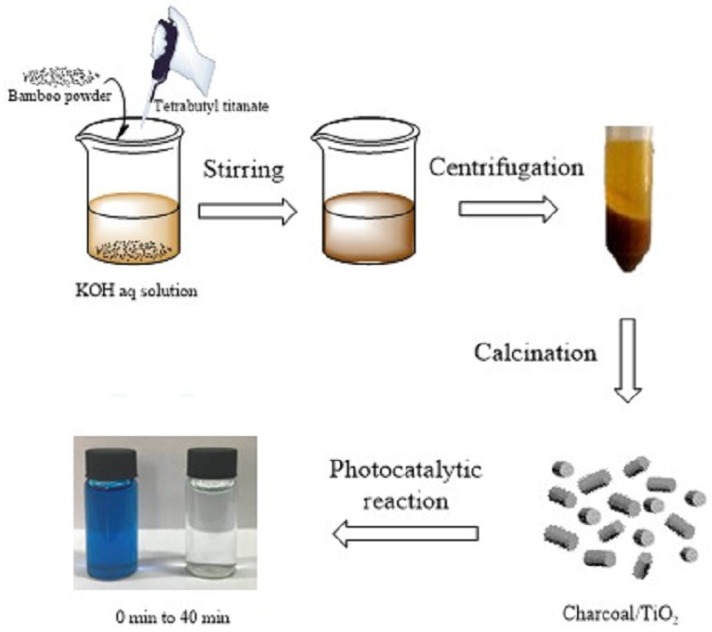
Schematic image of the preparation and photocatalytic property of bamboo charcoal/TiO_2_ composites.

The bamboo was cut into cylindrical materials, which have diameter of 5 mm and a length of 5–10 cm. The table-typed high speed crusher was used to crush the bamboo into powders with different sizes. The powders with different sizes, including the 0.425–0.185 mm (40–80 mesh), 0.185–0.125 mm (80–120 mesh), 0.125–0.098 mm (120–160 mesh), and less than 0.098 mm (>160 mesh) were applied as experimental materials. For the synthesis of bamboo-based biochar/TiO_2_ composites, different sizes of bamboo powder (2 g) and tetrabutyl titanate (0.68 g) were added into the KOH solution (30 mL, 3 mol L^−1^). Then, the as-mixed solution was stirring at room temperature for 1 h to obtain the homogeneous suspension. It obtained the precursors by centrifugal separation and washed with the deionized water followed by ethanol for three times, and dried at 60 °C for further characterization. The as-obtained precursors were designated as P-S1, P-S2, P-S3, P-S4, where -S1, -S2, -S3, and -S4 indicated the size (mm) from big to small. For example, P-S1 indicates the size of the bamboo power of 0.425–0.185 mm. For comparison, the sample was also synthesized by the similar method without tetrabutyl titanate using bamboo powder with the size of 0.125–0.098 mm. The dosage of other reagents and the other conditions are all kept the same. It was designated as P-S0. Finally, all of the as-obtained precursors were calcinated at 800 °C with the heating rate of 5 °C min^−1^, and was kept at this temperature for 3 h under the flowing nitrogen gas in the tube furnace. The as-obtained bamboo charcoal/TiO_2_ composites were designated as C-S1, C-S2, C-S3, C-S4, and C-S0. Figure 1 showed schematic image of the preparation and photocatalytic property of bamboo charcoal/TiO_2_ composites.

### 2.3. Characterization

Both the thermogravimetric (TG) and differential thermal gravity (DTG) curves were obtained at a heating rate of 10 °C min^−1^ in flowing nitrogen on a simultaneous thermal analyzer (DTG-60, Shimadzu, Japan). Scanning electron microscopy (SEM) was recorded on a Hitachi 3400 N scanning electron microscopy (Hitachi, Tokyo, Japan). Fourier-transform infrared (FT-IR) spectroscopic measurements were carried out with the wavenumber range from 4000 to 400 cm^−1^, at 64 scans per sample on Bruker VERTEX 70V (Karlsruhe, Germany). X-ray powder diffraction (XRD) patterns were recorded using a Rigaku D/Max 2200-PC X-ray diffractometer with high-intensity Cu Ka radiation (*λ* = 0.15418 nm) and a graphite monochromator. The UV-vis absorption spectra were taken on a UV 2450 spectrophotometer (Shimadzu, Japan).

### 2.4. Photocatalytic Experiment

Photocatalytic experiment of the samples was done using the photochemical reaction analyzer (Shanghai Bilong Instrument Manufacturing Co., Ltd., Shanghai, China). By dissolving the dye in distilled water, it obtained the methylene blue (MB) solution (12.8 mg L^−1^). The sample of 10 mg was then dispersed into the MB solution of 50 mL. In order to achieve the adsorption-desorption equilibrium, it stirred the resultant mixture for 30 min in the dark condition. It conducted the decomposition rate of MB in water under both ultraviolet light (mercury lamp, 700 W, Shanghai Bilong Instrument Manufacturing Co., Ltd., Shanghai, China) and visible light (xenon lamp, 700 W, Shanghai Bilong Instrument Manufacturing Co., Ltd., Shanghai, China) irradiation. The UV-vis spectrometer was applied to analyze the concentration of MB.

## 3. Results and Discussion

**Figure 2:**
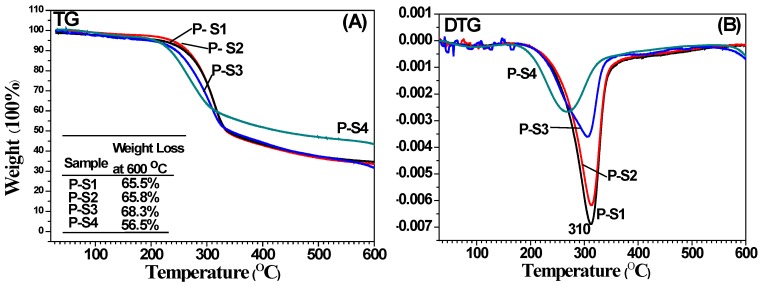
(**A**) Thermogravimetric (TG) curves and (**B**) differential thermal gravity (DTG) curves of bamboo charcoal-based composites precursors.

The thermal behavior of the precursors was investigated with TG and DTG under nitrogen atmosphere. As shown in Figure 2A, all of the samples contained about 2.7% of absorbed water (the weight loss below 100 °C), and the total weight losses of the bamboo power composites were 65.5%, 65.8%, 68.3%, and 56.5% for P-S1, P-S2, P-S3, and P-S4, respectively. These results indicated that with the decreased size of bamboo powders, the total weight losses slightly increased first then decreased. The initially degradation temperature was 240 °C for all of the samples. For the samples of P-S1, P-S2, and P-S3, the weight loss was mainly attributed to the transformation of bamboo power to carbon (Figure 2B), according with the endothermic peak located at 310 °C. The endothermic peak for P-S4 located at 265 °C. In general, the carbon-based samples showed the maximum weight loss around 580 °C. In this article, one can observe a loss peak 265 °C due to the existence of cellulose, hemicellulose, and lignin. These results indicated that the samples that were prepared using bamboo power with different sizes had different thermal stabilities.

**Figure 3:**
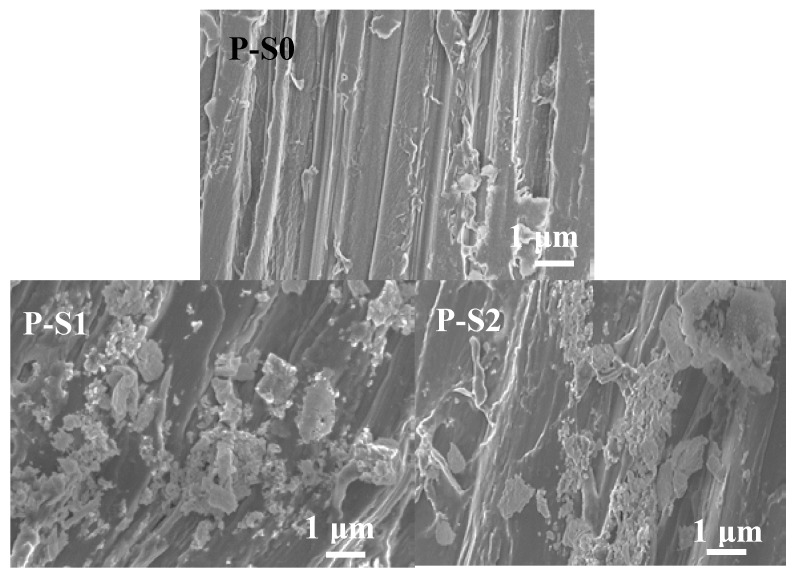

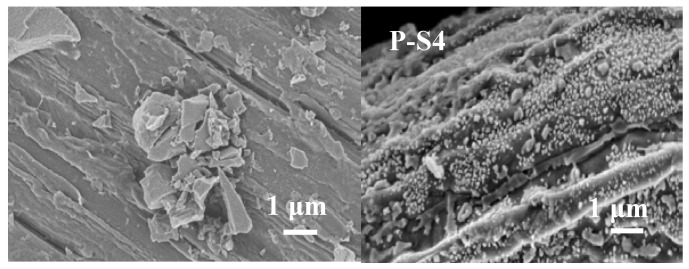
Scanning electron microscopy (SEM) images of P-S0, P-S1, P-S2, P-S3, and P-S4.

SEM measurements were applied to characterize the microstructures and morphologies of the precursors. Bamboo powder has a two-dimensional (2D) structure and many thin wrinkles were dispersed on the surface (Figure 3(P-S0)). In the previous study, these wrinkles were reported to induce the form of nano-channels on the surface of bamboo powder materials, which could transport electrons to suppress the recombination of photo-excited electron-hole pairs [21]. When the bamboo powder was used as the matrix, one can see that a large amount of TiO_2_ particles were dispersed on the surfaces of bamboo powder, as shown in Figure 3(P-S1 to P-S4). It was observed that the TiO_2_ particles were aggregated to form an irregular structure (Figure 3(P-S1 to P-S3)). When the size of the bamboo powder was reduced to 0.096 mm, one can see that the quantity of uniform TiO_2_ particles dramatically increased (Figure 3(P-S4)).

**Figure 4:**
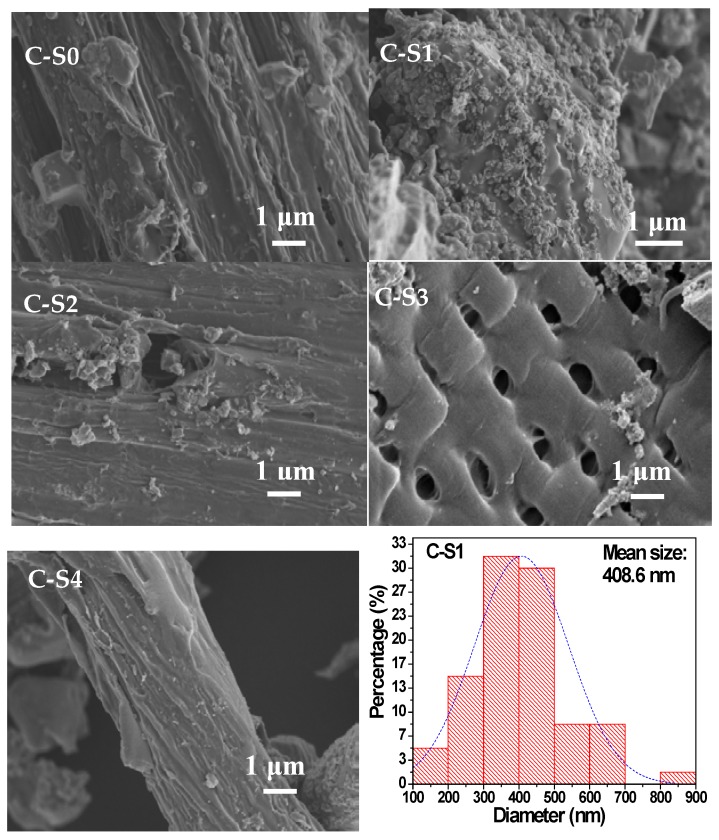
SEM images of C-S0, C-S1, C-S2, C-S3, C-S4, and the size distribution of C-S1.

SEM measurements were also conducted to characterize the microstructures and the morphologies of the samples after calcination. One can find that, after calcining, the surface of the bamboo charcoal still existed thin wrinkles and generated the small pore structure (Figure 4(C-S0)). As shown in Figure 4(C-S1), TiO_2_ particles were dispersed on the surface of bamboo charcoal. The mean size of TiO_2_ particles generated in the experiments is 408.6 nm, and the aperture of bamboo charcoal are mainly concentrated in about 2.27 μm (the size distribution of C-S1). Pore diameter of bamboo charcoal is about five times as much as particle size of TiO_2_ particles. As shown in both Figure 4(C-S2) and Figure 4(C-S3), TiO_2_ particles were intensively distributed on the surface and the edge of macroporous in the bamboo charcoal, which piled into irregular structure and did not block the pore structure of bamboo charcoal. But the quantity of TiO_2_ particles remarkably decreased. Based on these results, one can conclude that the size of bamboo had influence on the morphology of bamboo charcoal/TiO_2_ composites.

**Figure 5:**
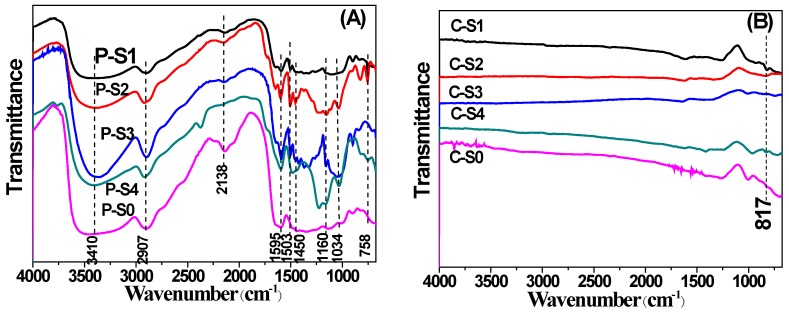
Fourier-transform infrared (FT-IR) spectra of (**A**) bamboo charcoal-based composites precursors of P-S1, P-S2, P-S3, P-S4, and P-S0; and (**B**) bamboo charcoal/TiO_2_ composites of C-S1, C-S2, C-S3, C-S4, and C-S0.

FT-IR spectra were conducted to study the functional groups of the as-obtained samples. The FT-IR spectra of precursors of bamboo charcoal-based composites, which were prepared at room temperature for 1 h, were shown in Figure 5A. It observed the characteristic peaks of cellulose, hemicellulose, and lignin. The stretching vibration in OH group of hemicellulose was observed at ~3410 cm^−1^. The characteristic peaks of hemicellulose were also observed at ~1450, 1160, and 1034 cm^−1^ in the spectrum. The shoulder at ~1160 cm^−1^ belongs to the arabinosyl side chains. The characteristic peak of all the C-O, C-C stretching, and the glycosidic (C-O-C) located at ~1034 cm^−1^ [22]. In the literature, it obtained the characteristic peak of the C-O stretching mode of cellulose at ~1034 cm^−1^ [23]. The peak at ~2897 cm^−1^ is due to the C-H stretching in CH_2_ and CH_3_ groups of cellulose, which is unaffected by changes of crystallinity. The peaks at ~1503 and ~1595 cm^−1^ indicated the aromatic skeletal vibration of lignin. It observed the absorbance peak of Ti-O that was stretching at 758 cm^−1^ [24].

The bamboo charcoal/TiO_2_ composites were obtained using the precursors by calcinated at 800 °C for 3 h under the flowing nitrogen gas. Figure 5B showed the FT-IR spectra of as-obtained bamboo charcoal/TiO_2_ composites samples. In comparison with Figure 5A, it observed the dramatically decreased intensities of peaks at 3410, 2897, and 1595 cm^−1^, thus demonstrating the degraded bamboo powder during the calcining process. In addition, it observed the absorption band of Ti-O stretching at 817 cm^−1^ for the bamboo charcoal/TiO_2_ composites, implying the as-fabricated bamboo charcoal/TiO_2_ composites by this method.

**Figure 6:**
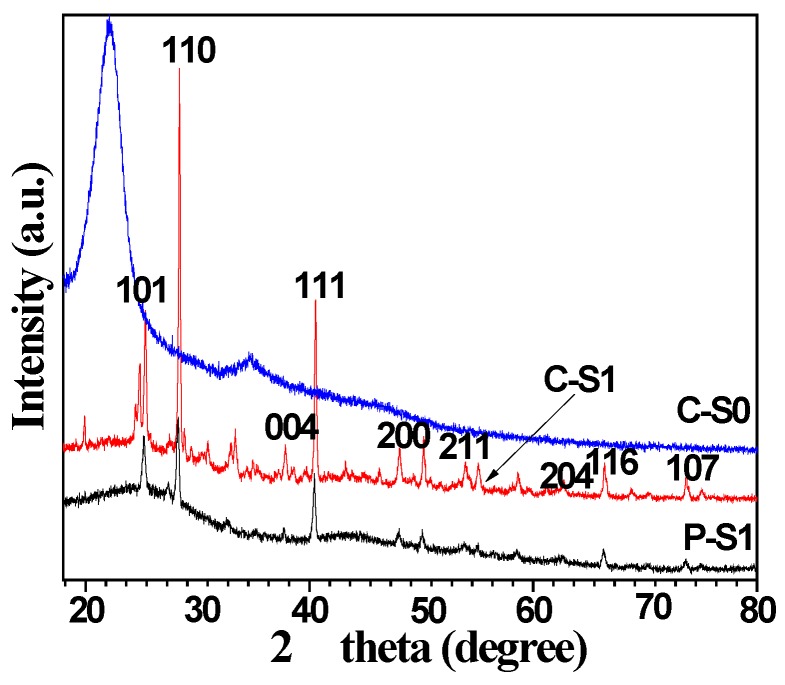
XRD patterns of C-S0, C-S1, and P-S1.

X-ray analysis provides the information about the crystal phase of the as-obtained materials. In the literature, it reported the greatly affected photocatalytic activity by the phase and crystallinity of the catalysts [12]. Figure 6(C-S0) displayed the XRD pattern of bamboo charcoal composites without adding tetrabutyl titanate. One can clearly observe a characteristic diffraction peak of cellulose. It is interesting to find that the bamboo charcoal/TiO_2_ composites are composed of the mixed phases of anatase and rutile, as shown in Figure 6(C-S1) and Figure 6(P-S1). Tetrabutyl titanate and the water reacted and fabricated TiO_2_ crystal nucleus by hydrolysis and the condensation reaction in this system. Reaction equations are listed, as follows:

Ti(OC_4_H_9_)_4_ + 4H_2_O → Ti(OH)_4_ + 4C_4_H_9_OH (1)

Ti(OH)_4_ + Ti(OC_4_H_9_)_4_ → TiO_2_ +4C_4_H_9_OH (2)

2Ti(OH)_4_ → 2TiO_2_ + 4H_2_O (3)

In order to reduce the free energy of the particle in the surface, TiO_2_ crystal nucleus tend to spontaneously reunite to form the aggregate, which gradually grew up because of Gibbs-Thomson effect. Eventually, TiO_2_ nanoparticles formed. In the XRD pattern of the precursor P-S1, it observed the six peaks of TiO_2_ at 2θ = 25.35, 37.91, 48.05, 62.76, 68.43, and 74.05, indexed to the (101), (004), (200), (204), (116), and (107) planes of TiO_2_, respectively (JCPDS No. 99-0008). It observed three peaks of TiO_2_ at 2θ = 28.19, 40.90, and 54.37, which were indexed to the (110), (111), and (211) planes of the rutile phase of TiO_2_. Calcinated the precursors at 800 °C for 3 h under the flowing nitrogen gas, the peaks intensities of TiO_2_ were obviously increased, implying the increase crystallinity of TiO_2_. In the literature, it reported the phase transformation of anatase to rutile at around 600 °C [25]. In this article, 800 °C was chosen as the calcination temperature. The crystallinity of the bamboo charcoal/TiO_2_ composites was 93.2%, which is enhanced remarkably, when compared with that of precursor (78.3%).

**Figure 7:**
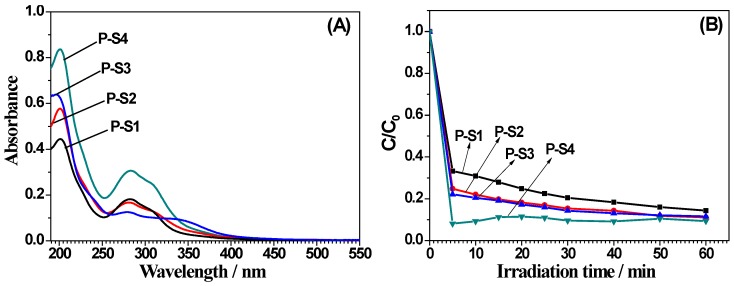
(**A**) UV-vis spectra and (**B**) photocatalytic degradation of MB under UV irradiation of bamboo charcoal-based composites precursors of P-S1, P-S2, P-S3, and P-S4.

Figure 7A is UV-visible absorption spectrum of bamboo charcoal-based composites precursors with different sizes. For all of the samples, the absorption is significantly enhanced at a wavelength less than 400 nm region due to TiO_2_ intrinsic absorption band (3.2 eV). With the decreasing of particle sizes, peak intensity gradually increased, and the absorption peak moved towards long wave, which was conducive to the absorption of visible light. Figure 7B is photocatalytic degradation of MB under UV irradiation by the bamboo charcoal-based composites precursors. One can easily see that with the decreasing of particle sizes, photocatalytic degradation rate of MB gradually enhanced, indicating that particle sizes played an important role in the photocatalytic activity.

**Figure 8:**
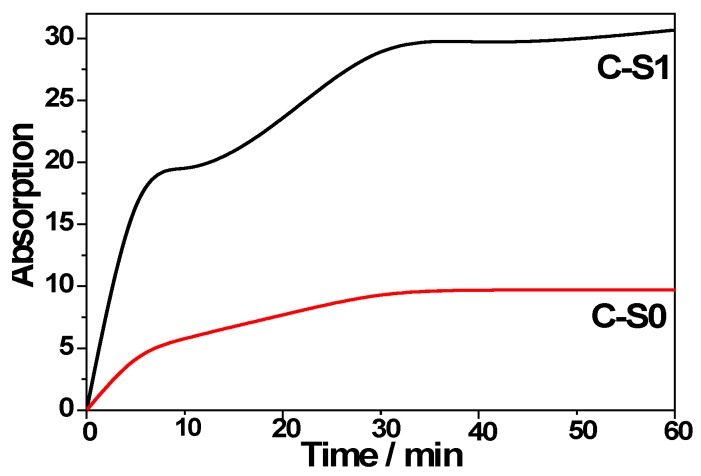
The adsorption rates of methylene blue (MB) on the different samples of C-S0 and C-S1.

The adsorption experiment was performed to evaluate the adsorption ability of C-S1 and C-S0 in the dark by UV-vis spectrum. The adsorption rate curves of MB on the composites were investigated, as shown in Figure 8. After equilibration for 60 min, 30.7% of MB was removed from the solution C-S1 and 9.7% of MB was removed from the solution of C-S0, respectively. For the sample of C-S0, there was a rapid adsorption within the first 10 min, it slowly leveled off, and then reached adsorption equilibrium in 30 min. For the sample of C-S1, there was a rapid adsorption within the first 30 min, then slowly leveled off, and reached to adsorption equilibrium in 60 min. When compared with the sample of C-S0, the adsorption ability of MB in the CS1 composite increased due to the increase of specific surface area (Table 1). These results indicated that the as-obtained composites displayed the enhanced absorptive capacity.

**Figure 9:**
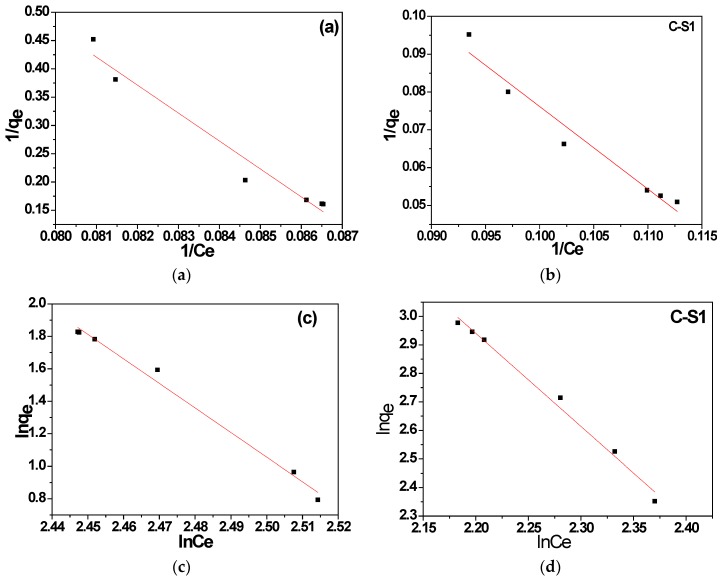
(**a**,**b**) Langmuir adsorption isotherm and (**c**,**d**) Freundlich adsorption isotherm for MB adsorption of (**a**,**c**) C-S0 and (**b**,**d**) C-S1.

The equilibrium adsorption about MB was conducted using the adsorption isotherms, such as Langmuir and Freundlich isotherm models. It reported a homogeneous system of the Langmuir isotherm and a heterogeneous system of Freundlich isotherm [23]. Figure 9 displayed the adsorption data. It found little high correlation coefficients for the Freundlich isotherm model (R^2^ = 0.988, 0.989), when compared with that for the Langmuir isotherm model (R^2^ = 0.952, 0.957).

**Figure 10:**
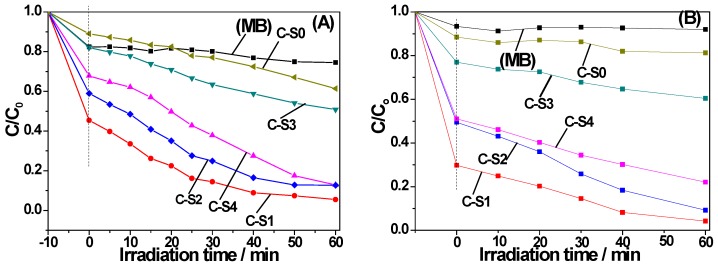
Photocatalytic degradation of MB using the samples (**A**) under UV irradiation and (**B**) under visible light irradiation.

By measuring the decomposition rate of MB in water under both ultraviolet light and visible light irradiation, it evaluated the photocatalytic activity of bamboo charcoal/TiO_2_ composites. Figure 10A displayed the photocatalytic activity of as-obtained composites with different sizes under the UV irradiation. The sample without catalysts was used as control (Figure 10A). Bamboo charcoal/TiO_2_ composites showed higher catalytic activity than that without TiO_2_. After the UV irradiation for 60 min, the degradation rates were 95%, 87%, 49%, and 86% for C-S1, C-S2, C-S3, and C-S4, respectively. The nitrogen adsorption-desorption isotherms results for the samples are listed in Table 1. It was found that the surface area decreased with the decreasing sizes of bamboo charcoal. The pore volume also presented a similar result. Both the porous structure and the high surface area are beneficial for reactant adsorption. Therefore, with the decrease size of bamboo charcoal, photocatalytic degradation efficiency decreased. When the size is less than 0.096 nm, the photodegradation efficiency gradually improved. Figure 10B exhibited the photocatalytic activity of as-obtained composites with different sizes under visible light irradiation. In the visible light catalytic after 60 min, the degradation rates were 97%, 91%, 40%, and 78% for C-S1, C-S2, C-S3, and C-S4, respectively. Photocatalytic activity of bamboo charcoal/TiO_2_ decreased first then increased with the decreasing size of bamboo charcoal.

**Figure 11:**
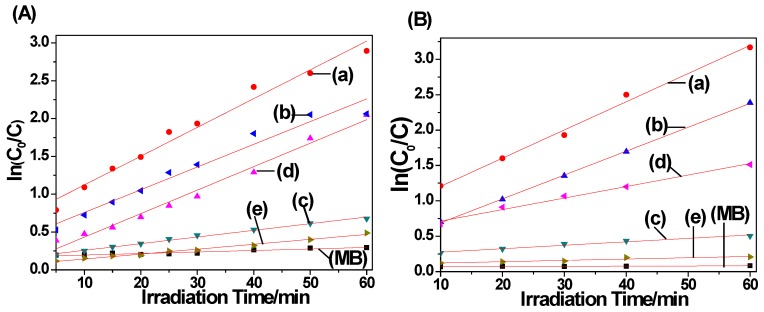
Linear transform ln(C_0_/C) = f(t) of the kinetic curves of MB (**A**) under UV irradiation and (**B**) under visible light irradiation: (a) C-S1, (b) C-S2, (c) C-S3, (d) C-S4, and (e) C-S0.

It observed the photocatalytic reaction following pseudo-first-order kinetics ln(C_0_/C) = kt using MB with low concentration, where C_0_ is the initial concentration of MB solution after adsorption equilibrium, C is the MB concentration at any time t, k is on behalf of the kinetic constant. The equation was applied to simulate the light catalytic data, explaining the reaction kinetics of the MB degradation. Under the UV irradiation, it observed the relative coefficient R^2^ of the fitting curves of 0.976, 0.963, 0.992, 0.983, 0.987, and 0.896 for C-S1, C-S2, C-S3, C-S4, C-S0, and MB, respectively. The values are 0.991, 1.000, 0.968, 0.987, 0.857, and 0.798 for C-S1, C-S2, C-S3, C-S4, C-S0, and MB under the visible light irradiation. Before the UV irradiation, it observed the value of total organic carbon (TOC) of 19.32 mg L^−1^ for MB. After the UV irradiation, it observed the decrease value of TOC with the charcoal/TiO_2_ nanocomposites. Therefore, one can conclude the photodegradation reactions following the pseudo-first-order kinetics (Figure 11). Meanwhile, the kinetic constants decreased first, then increased with decreasing bamboo charcoal sizes. These results indicated that the photocatalytic activity of bamboo charcoal/TiO_2_ composites decreased first then increased with the decreasing size of bamboo charcoal.

**Figure 12:**
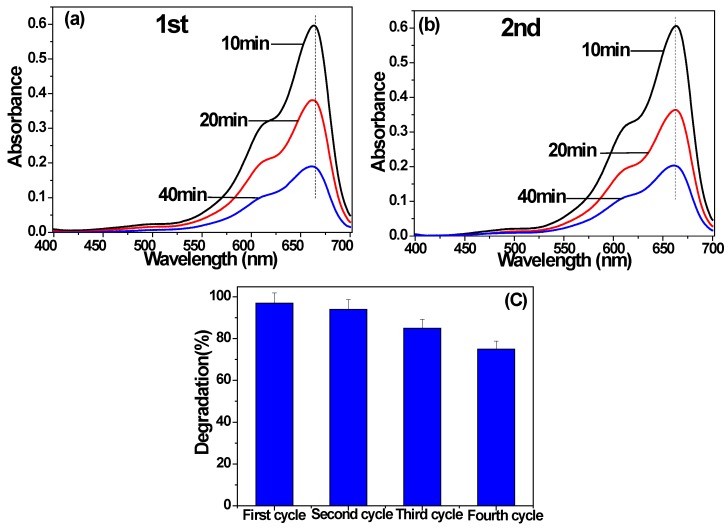
Absorption spectra of MB solution at different periods of the time in the presence of C-S1 under visible light in (**a**) the first cycle and (**b**) the second cycle; (**c**) photocatalytic degradation of MB after up to four cycles measured after visible light irradiation of duration 240 min.

In order to examine the stability of photocatalytic performance of bamboo charcoal/TiO_2_ composites, repeatability test of the sample was done. Figure 12a,b displayed the absorption spectra of MB solution under the visible light irradiation in the presence of C-S1 in the first and second cycle. One can see that with the increase of time, maximum absorbance of MB decrease constantly. In the second circulation, the degradation rate of MB almost unchanged, when compared with that of the first time.

Figure 12c is photocatalytic degradation of MB after up to four cycles measured after the visible light irradiation of duration 240 min. After the bamboo charcoal/TiO_2_ composites were used for four times, the photocatalytic degradation rate was 75%. While the degradation rate kept stability as compared with the first cycle (97%). Further study showed that the bamboo charcoal/TiO_2_ composites are easy to be removed and re-used many times.

**Figure 13:**
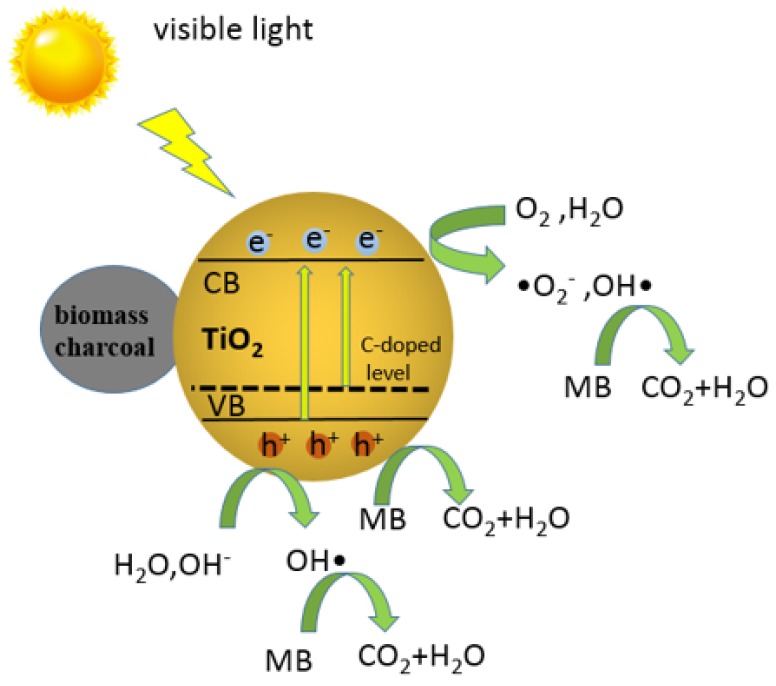
The schematic diagram of photocatalytic degradation of MB over bamboo charcoal/TiO_2_ nanocomposites.

Based on the above study, one speculates the feasible mechanism of photocatalytic degradation over bamboo charcoal/TiO_2_ composites, as following: In the bamboo charcoal/TiO_2_ composites, bamboo charcoal itself displayed no catalytic effect, which was used matrix for the synthesis of bamboo charcoal/TiO_2_ composites with large specific surface area. These structures are advantageous for the improve photocatalytic reaction on the bamboo charcoal/TiO_2_ composites. As shown in the Figure 13, under visible light irradiation, the conduction band and valence band of TiO_2_ generate, respectively, electrons and holes; the photoproduction hole of valence band has a strong oxidizing. It can put the water and hydroxyl oxidatize into hydroxyl radicals. The generated hydroxyl radicals can also oxidatize and decompose pollutants. At the same time, electronics of conduction band can react with oxygen and water to generate hydroxyl free radicals and the superoxide free radicals and other strong oxidizing species. •O_2_^−^, OH•, and holes decompose pollutants into CO_2_ and H_2_O. Bamboo charcoal also reinforced adsorption the ability of composites to MB, promoting the proceed of photocatalytic reaction on the surface of composites.

## 4. Conclusions

In summary, the bamboo charcoal/TiO_2_ composites were successfully prepared by the calcination method using bamboo powder with different sizes as the carbon source. The different size of bamboo powder had an effect on the morphology and the crystallinity of composites. The influences of size of bamboo charcoal on the synthetic products were also explored. The bamboo charcoal/TiO_2_ composites displayed the improve photocatalytic activities under the visible light irradiation and UV irradiation. The adsorption isotherms better fitted Freundlich isotherm models and the photodegradation reactions followed pseudo-first-order kinetics. Bamboo charcoal/TiO_2_ composites also exhibited high stability after up to four cycles. Carbon doping by other organic polymers (e.g., cellulose, hemicellulose, lignin, etc.) may provide a green strategy to improve the photocatalytic property of TiO_2_.

## Figures and Tables

**Table 1:** S_BET_, total pore volume for C-S1, C-S2, C-S3, C-S4, and C-S0.

Sample Name	S_BET_ (m^2^/g)	Total Pore Volume (cm^3^/g)
C-S1	258.09	0.16
C-S2	93.10	0.07
C-S3	5.82	0.03
C-S4	12.76	0.05
C-S0	5.37	0.02
